# Effects of cardiac output-guided hemodynamic management on fluid administration after aneurysmal subarachnoid hemorrhage

**DOI:** 10.1186/cc13645

**Published:** 2014-03-17

**Authors:** B Bergmans, M Egal, J Van Bommel, J Bakker, M Van der Jagt

**Affiliations:** 1 Erasmus MC - University Medical Center, Rotterdam, the Netherlands

## Introduction

In patients with aneurysmal subarachnoid hemorrhage (SAH), hypervolemic therapy may result in fluid overload that may be associated with adverse clinical outcomes [1,2]. We hypothesized that a goal-directed transpulmonary thermodilution (TPT) monitoring protocol aiming for normovolemia may result in decreased fluid intake while sustaining adequate volume status in poor-grade SAH patients.

## Methods

Following the introduction of the hemodynamic protocol in 2011, 26 consecutive patients with SAH were included until 2013 Using TPT (PiCCO; Pulsion), cardiac output (CO), global end- diastolic volume index (GEDVI) and extravascular lung water index (EVLWI) were determined. Fluid administration was targeted at fluid unresponsiveness. Indications for start of the protocol were: hypotension (in spite of fluids), pulmonary edema or cardiac stunning, daily fluid balance ≤-1 l, cerebral ischemia (DCI) with progressive symptoms. Data were collected on fluid intake and output up to 3 days before and 3 days after the start of TPT. We assessed the course of fluid input and output and hemodynamic parameters before and after the start of the protocol with the generalized estimating equation.

## Results

Mean age was 55 ± 16, and median Glasgow Coma Scale on admission was 8 (IQR 6 to 13). TPT was started at a median of 1 day after ICU admission (IQR 0 to 4). DCI developed in 70% and the in-hospital death rate was 45%. Compared with days preceding the protocol (day -3 to -1), TPT (day 0 to 3) was associated with decreased fluid intake (compared with day 3 as reference; day -1: +1.14 ± 0.31 l, *P *< 0.001; day 0: +0.68 ± 0.29 l, *P *= 0.019; day 1: +0.73 ± 0.31 l, *P *= 0.02), increased fluid output (day -2: -0.91 ± 0.46 l, *P *< 0.05 compared with day 3), and consequently a strong decrease in fluid balance (day -3: +2.10 ± 0.56 l *P *< 0.001; day -2: +2.66 ± 1.14 l, *P *= 0.02; day -1: +1.41 ± 0.37 l, *P <*0.001). The decreased fluid intake and fluid balances did not result in decreased CO, GEDVI or EVLWI on days 1 to 3 compared with day 0 (mean PCCI 3.5 l/minute/m^2^, mean GEDVI 761 ml/m^2^, mean EVLWI 10.8 ml/kg).

## Conclusion

Our data suggest that in poor-grade SAH patients goal- directed fluid management with TPT is feasible to decrease intake and increase diuresis without adverse effects on cardiac output or preload parameters. Future research should assess the effect of such a protocol on clinical outcomes.
